# Bright PD-1 expression by flow cytometry is a powerful tool for diagnosis and monitoring of angioimmunoblastic T-cell lymphoma

**DOI:** 10.1038/s41408-020-0301-x

**Published:** 2020-03-06

**Authors:** Mariko Yabe, Qi Gao, Neval Ozkaya, Sarah Huet, Natasha Lewis, Janine D. Pichardo, Alison J. Moskowitz, Steven M. Horwitz, Ahmet Dogan, Mikhail Roshal

**Affiliations:** 1 Hematopathology Service, Department of Pathology, Memorial Sloan Kettering Cancer Center, New York, NY USA; 2 Laboratory of Pathology, Center for Cancer Research, National Cancer Institute, Bethesda, MD USA; 3 Hospices Civils de Lyon, Centre Hospitalier Lyon Sud, Laboratoire d’Hématologie, Pierre-Bénite, France; 4 Cancer Research Center of Lyon, INSERM U1052 UMR CNRS 5286, Equipe labellisée LIGUE Contre le Cancer, Lyon, France; 5 Lymphoma Service, Department of Medicine, Memorial Sloan Kettering Cancer Center, New York, NY USA

**Keywords:** T-cell lymphoma, Diagnosis

Angioimmunoblastic T-cell lymphoma (AITL) is one of the most common specific types of peripheral T-cell lymphomas (PTCL)^1^. The cell of origin for AITL is T follicular helper cells (TFH)^2,3^. AITL has an immunophenotype closely akin to that of normal TFH; CD4+, CD8−, T-cell receptor (TCR) alpha/beta, and often expressing CXCL13, CD10, BCL6, Programmed-Death-1 (PD-1), ICOS, and CD200^2,4–6^. Diagnosis of AITL is challenging especially in small needle core biopsy due to the presence of admixed abundant inflammatory cells. The differential diagnosis is broad, and a definitive diagnosis requires a use of ancillary testing, including large panels of immunohistochemistry and molecular genetic studies. Immunophenotyping by flow cytometry is a common study for the diagnosis as well as for monitoring minimal residual disease of hematologic malignancies^7–13^, but contribution of flow cytometry to diagnose AITL, while often reported, has been unclear due to lack of systemic studies with integrated morphologic assessment^14^. Here, we evaluated the utility of highly sensitive flow cytometry in diagnosing AITL using an antibody against PD-1 in the context of other T-cell antigen. Tissue biopsies, bone marrow biopsies, and peripheral blood samples were retrieved from pathology archives between May 2015 and December 2018 at Memorial Sloan Kettering Cancer Center. This study was performed following the declaration of Helsinki and was approved by the institutional review board. The group was composed of 81 patients with PTCL and 40 patients with no T-cell lymphomas, but with potential morphologic mimics. Specifically, 36 patients with AITL, 6 with ALK-negative anaplastic large cell lymphoma, 2 with ALK-positive anaplastic large cell lymphoma, 9 with adult T-cell leukemia/lymphoma, 11 with peripheral T-cell lymphoma, not otherwise specified, 6 with nodal involvement by mycosis fungoides, 5 with T-cell large granular lymphocytic leukemia, 6 with T-cell prolymphocytic leukemia, 4 with nodular lymphocyte-predominant Hodgkin lymphoma, 1 with T-cell/histiocyte-rich large B-cell lymphoma, 5 with diffuse large B-cell lymphoma, germinal center B-cell type, 6 with follicular lymphoma, 12 with classical Hodgkin lymphoma, and 12 with benign reactive lymph nodes. Overall we obtained 94 tissue biopsies, 59 peripheral blood specimens, and 21 bone marrow aspirate specimens (Supplementary Table 1). Expression of PD-1 (CD279) on lymphoma cells was evaluated by BD FACSCanto 10-color flow cytometry (BD Biosciences, San Jose, CA) with BV605-conjugated anti-CD279 antibody (EH12.2H7, BioLegend, San Diago, CA) along with other T-cell antigens frequently evaluated in this context. The results were analyzed with Woodlist software (Dr. B.L.Wood, University of Washington). Abnormal T-cell populations were identified by visual assessment of aberrant antigen expression. Mean fluorescence intensity (MFI) of anti-CD279 antibody was also evaluated. For selected cases, Beta Mark TCR Vbeta Repertoire Kit (Beckman-Coulter, Miami, FL) was used to confirm clonality. Flow sorting was performed in conjunction with molecular genetic analysis as a part of the study. Detailed methods are described in supplementary information.

Flow cytometric analysis of all 12 reactive lymph nodes demonstrated that non-neoplastic T-cell population with bright PD-1 (CD279) uniformly co-express CXCR5 (CD185) and ICOS (CD278), consistent with TFH (Fig. 1a–f). No other physiological subset of normal T-cell showed PD-1 expression with similar intensity indicating that bright PD-1 expression is a hallmark of TFH. These findings were reproducible in neoplastic T-cells of AITL and confirmed that bright PD-1 expression is an excellent surrogate to assign TFH phenotype to T-cells (Fig. 1g–i). Subsequently, we compared the expression levels of PD-1 by flow cytometry among PTCL cases. The expression of PD-1 in abnormal T-cell population in the tissue biopsy was significantly higher in AITL than that of other PTCL (CD279-BV605 MFI; Mean ± SEM; 6990.0 ± 934.9 vs. 547.3 ± 155.5; *p* < 0.0001) (Fig. 1j). PD-1 MFI differentiated AITL from other PTCL with high sensitivity and specificity (AUC 0.978; Fig. 1k). In 54 patients with PTCL who had circulating lymphoma cells in peripheral blood, the significant increase in PD-1 expression was a consistent finding and it differentiated AITL from other PTCL with high sensitivity and specificity (CD279-BV605 MFI; Mean ± SEM; 3079.0 ± 508.8 vs. 341.8 ± 54.17; *p* < 0.0001, AUC 0.986, Fig. 1l, m). Among 28 AITL patients with a tissue biopsy, 14 patients had concomitant peripheral blood samples. Circulating AITL cells were detected in all 14 patients, including 7 patients with new diagnosis of AITL and 7 patients with relapsed/persistent AITL (median 0.52% of total white blood cells; Range 0.094–7.7%). ddPCR for selected mutations was performed on flow sorted PD-1 bright T-cell population in peripheral blood and bone marrow in 3 and 2 patients with AITL, respectively, and same genetic abnormalities seen in tissue sample were identified, confirming clonal identity between tissue and peripheral blood/bone marrow. These findings suggest that peripheral blood flow cytometry with anti-PD-1 antibody is a useful screening tool for diagnosis of AITL. Aberrant T-cells with bright PD-1 expression were not identified in bone marrow or in peripheral blood of the patients with B-cell lymphoma or reactive follicular hyperplasia.

**Fig. 1:**
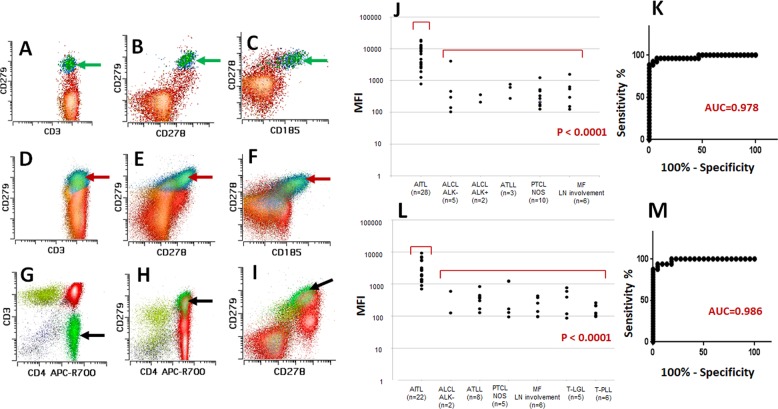
Flow cytometric findings of PD-1 (CD279), ICOS (CD278), and CXCR5 (CD185) expression in non-neoplastic T-cells and T-cell lymphoma. Non-neoplastic T-cell population with bright PD-1 expression in reactive lymph node (green arrow) shows co-expression of ICOS and CXCR5 (**a**–**c**). Non-neoplastic T-cell population with bright PD-1 expression in lymph node with follicular lymphoma (red arrow) shows co-expression of ICOS and CXCR5 (**d**–**f**). Abnormal CD4-positive T-cell population with loss of surface CD3 identified in lymph node with AITL (**g**, black arrow; Case 11). This abnormal T-cell population shows bright PD-1 expression (**h**). This population also co-express ICOS and CXCR5 (**i**, CD185 plot not shown). PD-1 mean fluorescent intensity (MFI) in tissue sample. MFI in AITL is significantly higher than that of non-AITL PTCL (**j**). Receiver Operating Characteristic (ROC) Curve of PD-1 MFI in tissue sample shows high sensitivity and specificity observed in this assay differentiating AITL from other PTCL (*p* < 0.001, AUC 0.978: sensitivity 93.15% and specificity 92.86% for a cutoff of MFI 1560.64) (**k**). PD-1 mean fluorescent intensity (MFI) in peripheral blood sample. MFI in AITL is significantly higher than that of non-AITL PTCL (**l**). ROC curve of PD-1 MFI in peripheral blood sample also shows high sensitivity and specificity in differentiating AITL from other PTCL (*p* < 0.001, AUC 0.986: sensitivity 93.75% and specificity 95.45% for a cutoff of MFI 836.33) (**m**).

All 28 AITL cases with tissue biopsy showed CD3 and PD-1 immunoreactivity by immunohistochemistry, and PD-1 expression on abnormal T-cells by flow cytometry showed excellent correlation with immunohistochemistry (Supplementary Table 2, Fig. 2a–i). In tissue samples, we frequently encountered that other standard TFH markers were not informative by immunohistochemistry, or interpretation of immunohistochemistry was challenging. In our cohort, expression of other TFH markers was not observed by immunohistochemistry in seven cases (25% of cases; Case #3, 7, 8, 12, 15, 19, 28). Additional three cases (11% of cases; Case #21, 22, 27) presented with concomitant lymphoid neoplasm, which obscured morphologic and immunohistochemical evaluation for AITL (Fig. 2j–o). In 10 patients (36% of cases), AITL could not be initially established by morphology and immunohistochemistry by expert review; however, flow cytometry reliably detected AITL in all these 10 challenging cases. Next-generation sequencing was performed with FFPE tissue on 14 AITL cases, including these challenging cases, and all cases had at least one mutation either in *RHOA*, *TET2*, *IDH2*, or *DNMT3A* (Supplementary Tables 3 and 4). Sorted bright PD-1 positive neoplastic T-cells in two cases of AITL with *RHOA G17V* mutation showed the presence of same mutation with over 0.45 allele frequency, confirming that this population represents AITL. We performed TCR Vbeta fragment analysis by flow cytometry on selected AITL cases when other phenotypic abnormalities were subtle. The abnormal PD-1 bright T-cell population showed restricted Vbeta usage indicating monoclonality (Fig. 2p–t). Monoclonality was also confirmed by molecular *TCR* analysis. None of the 6 patients with morphologic mimics with bright PD-1 expression which were tested for TCR Vbeta analysis showed evidence of monoclonality, and findings were also confirmed by molecular *TCR* analysis (Fig 2u–ad). All 28 cases of AITL showed at least one loss (negative or dim) of T-cell antigen by flow cytometry (Supplementary Table 2). Among 40 cases of morphologic mimics of AITL, T-cell antigens were preserved with exception of populations of CD4+ T cells showing reduction of CD7, commonly seen in memory T cells (Fig 2u–ad). In our study, TCR Vbeta restriction along with loss of T-cell antigen(s) in PD-1 bright T-cell population reliably discriminated morphologic mimics from AITL with 100% sensitivity and specificity. We also found that PD-1 flow cytometry is useful for evaluating staging bone marrow biopsy especially for the cases with minimal involvement or cases having other concomitant hematolymphoid malignancy (Supplementary Fig. 1).

**Fig. 2:**
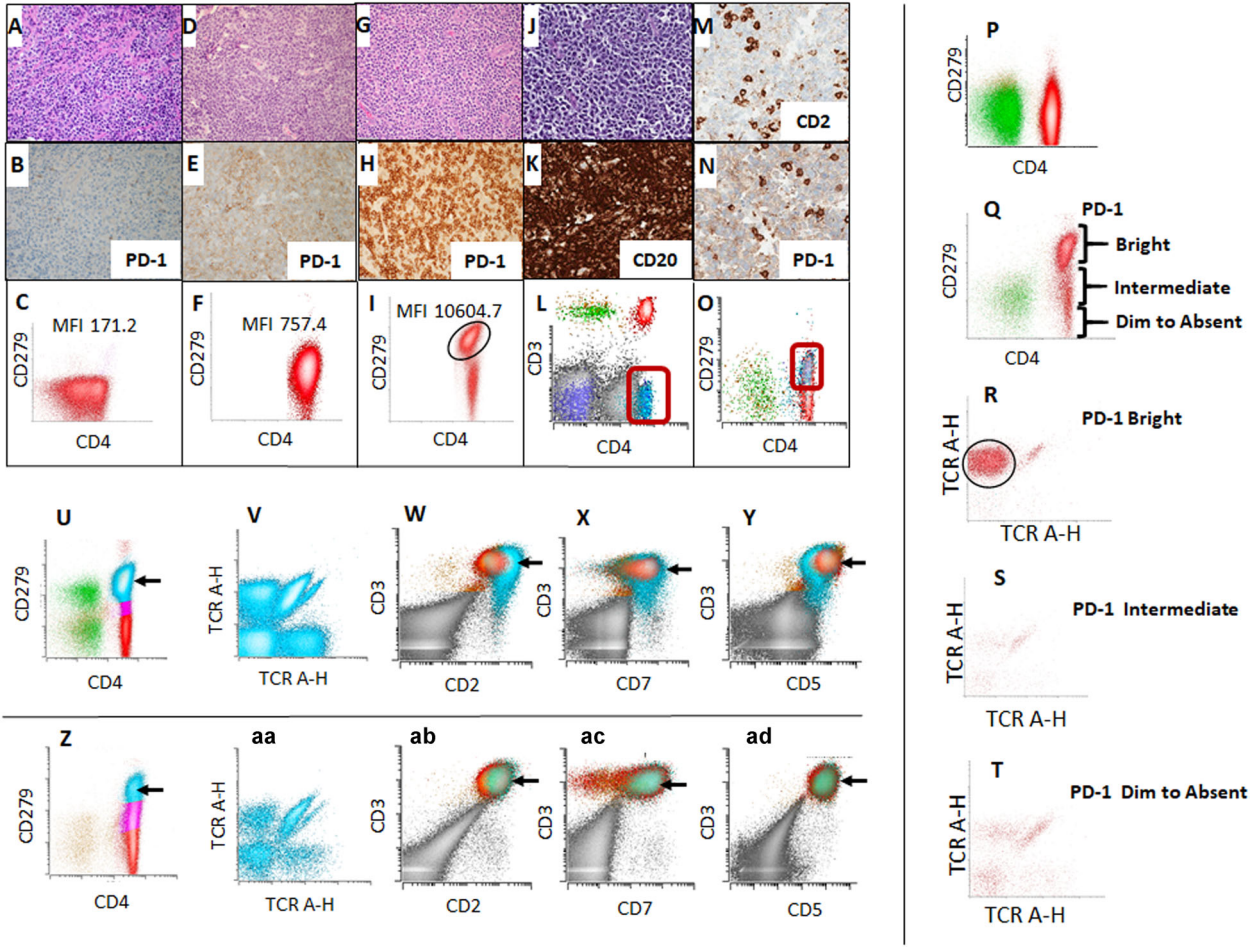
Utility of flow cytometry in diagnosing angioimmunoblastic T-cell lymphoma. Histology, PD-1 immunohistochemistry and PD-1 flow cytometric findings in peripheral T-cell lymphoma, NOS (PTCL-NOS; **a**–**c**), adult T-cell leukemia/ lymphoma (ATLL; **d**–**f**), and angioimmunoblastic T-cell lymphoma (AITL, Case 28; **g**–**i**). PD-1 expression is not observed in PTCL-NOS both by immunohistochemistry (**b**) and flow cytometry (**c**). ATLL shows intermediate expression of PD-1 by immunohistochemistry (**e**) and flow cytometry (**f**). AITL shows bright PD-1 expression by immunohistochemistry (**h**) and flow cytometry (**i**). Lymph node involved by both AITL and diffuse large B-cell lymphoma (Case 21; Fig 2j–o). Lymph node shows atypical lymphocytic infiltrate composed of large cells (**j**). Immunohistochemistry for CD20 highlights sheets of large cells (**k**). CD2 highlights scattered T-cells (**m**). Scattered T-cells also express PD-1 (**n**). Flow cytometry identifies abnormal CD4+ T-cell population with aberrant loss of surface CD3 (**l**; aqua dots). This aberrant population expresses PD-1/CD279 (**o**; aqua dots). PD-1 expression on normal lymph node and lymph node involved by AITL. Normal lymph node does not show distinct CD4+/PD-1 bright population (**p**). Lymph node involved by AITL show distinct CD4+/PD-1 bright population (**q**, Case 17). Vbeta fragment analysis performed on sorted PD-1 bright population shows that this population is clonal (**r**, Case 17). Vbeta fragment analysis performed on PD-1 intermediate and dim to absent populations show that these populations are polyclonal (**s**, **t**, Case 17). Flow cytometric findings of patients with nodular lymphocyte-predominant Hodgkin lymphoma (**u–y**) and reactive follicular hyperplasia (Z-AD). Prominent CD4-positive T-cell population with bright PD-1 expression is seen in NLPHL (**u**; blue dots). This population is polyclonal by Vbeta analysis (**v**). PD-1 bright population shows normal T-cell immunophenotype of CD2+/CD3+/CD4+/CD5+/CD7+(**w**–**y**; blue dots). Expansion of CD4-positive T-cell population with bright PD-1 expression is present in reactive follicular hyperplasia (**z**; blue dots). This population is polyclonal by Vbeta analysis (**aa**). PD-1 bright population shows normal T-cell immunophenotype of CD2+/CD3+/CD4+/CD5+/CD7+(**ab**–**ad**; blue dots). **a**, **d**, **g**, **j**: Hematoxylin and Eosin, ×400; **b**, **e**, **h**, **n**: PD-1 immunohistochemistry, ×400; **k**: CD20 immunohistochemistry, ×400; **m**: CD2 immunohistochemistry, ×400.

Previous studies identified abnormal T-cell population with sCD3-/CD4+immunophenotype in AITL^9–13^. In our cohort, ~15% of AITL show two distinct abnormal T-cell populations with and without surface CD3 expression (Case 10, 12, 17, 23). For these cases, in addition to the frankly abnormal T-cell population with loss of sCD3, an additional population with sCD3+/CD4+/PD-1 bright phenotype was revealed to be monoclonal by Vbeta fragment analysis and molecular *TCR* analysis (Supplementary Fig. 2). Our study demonstrated that the tumor burden could be highly underestimated when only surface CD3-negative population is considered. Co-existence of surface CD3 positive and negative populations within the same AITL is to our knowledge a novel finding that deserves further pathophysiologic investigation.

The current WHO classification recognizes other nodal lymphomas of TFH origin, including follicular T-cell lymphoma and nodal PTCL with TFH phenotype; that said this entity is rare, and the diagnosis likely has similar clinical implications to AITL^5^. Among our 28 cases, one case has a diagnosis of PTCL with TFH phenotype based on the morphological assessment (Case 26). Although case number is limited, our finding of bright PD-1 expression by flow cytometry was a consistent finding in PTCL with TFH phenotype.

In conclusion, flow cytometric immunophenotyping with anti-PD-1 antibody reliably detects neoplastic population in AITL including challenging and complex cases. Flow cytometric analysis reliably differentiates AITL from other PTCL and morphologic mimics with high sensitivity and specificity. Peripheral blood flow cytometry for PD-1 expression is a very useful tool in supporting or not supporting the diagnosis of AITL in corresponding tissue biopsy. Without exception, AITL appears to be a systemic (stage IV) disease at presentation when assessed with sensitive techniques, and this implies the utility of flow cytometry for peripheral blood-based screening for AITL. Measurement of PD-1 expression by flow cytometry is a powerful tool in diagnosis and monitoring of AITL.

## Supplementary information

Supplemental information
