# Supplementary material for: Bright PD-1 expression by flow cytometry is a powerful tool for diagnosis and monitoring of angioimmunoblastic T-cell lymphoma
Source: Blood Cancer J. 2020 Mar 6;10(3):32. doi: 10.1038/s41408-020-0301-x (PMC7060322; doi:10.1038/s41408-020-0301-x)
Supplement: Supplementary file 1 — Supplemental information [file 41408_2020_301_MOESM1_ESM.pdf]

Supplementary information

Methods

Flow cytometric analysis

The expression of PD-1 (CD279) on lymphoma cells was evaluated by BD FACSCanto 10-color flow cytometry (BD Biosciences, San Jose, CA) with BV605-conjugated anti-CD279 antibody (EH12.2H7, BioLegend, San Diago, CA) along with other T-cell antigens frequently evaluated in this context. Other antibodies for multiparameter flow cytometry were as follows; CD7-BB515 (M-T701, BD Horizon, San Jose, CA), CD56-PE (N901, Beckman-Coulter, Miami, FL), CD3-PC7 (UCHT1, Beckman-Coulter), CD5-APC (L17F12, BD Biosciences), CD4-APC-A700 (13B8.2, Beckman-Coulter), CD8-APC-H7 (SK1, BD Biosciences), CD2-BV421 (RPA-2.10, BD Horizon), CD45-V500C (2D1, BD Horizon), CD185 (CXCR13)-PE (MU5UBEE, eBiosciences, San Diego, CA), CD278 (ICOS)-APC (ISA3, eBiosciences). The results were analyzed with Woodlist software (Dr. B.L.Wood, University of Washington). An abnormal T-cell population was identified by visual assessment of population with aberrant antigen expression, and mean fluorescence intensity (MFI) of BV605-conjugated anti-CD279 antibody was evaluated in each case. For selected cases with TFH expansion, Beta Mark TCR Vbeta Repertoire Kit (Beckman-Coulter) was used to confirm clonality. Flow cytometric analysis of TCR Vbeta repertoire is a methodology that uses fluorescently labeled anti-TCR Vbeta antibodies to determine if the identified T-cell populations represent a diverse or a clonal process.[1-4] In original protocol, the assay is run in eight separate tubes with three Vbeta family-specific antibodies in each tube (one labeled with FITC, one labeled with PE, and one labeled

with FITC and PE). Through the use of gating reagents (for example CD3, CD4, or CD8), the T-cell population of interest can be isolated to determine if there is evidence for overrepresentation of a particular TCR Vbeta isoform relative to normal, a finding that is strongly suggestive of clonality. For this study, modified method was applied; instead of 8-tube analysis as intended by the manufacturer, all the fluorescently labeled TCR V-beta antibodies are combined into a single tube for combined analysis.[5]

Flow sorting

For selected cases, flow sorting was performed in conjunction with molecular analysis (described below). Samples were pre-lysed by 10% ammonium chloride lysing solution and stained using 10-color T-cell panel mentioned in above. The abnormal T-cell population was identified and tightly gated by its aberrant immunophenotype. Cell sorting was performed on BD FACS Aria Fusion (BD Biosciences) by four-way sorting using an 85 um nozzle. The abnormal T-cell population was sorted into RPMI-1640 (Corning Inc. Corning, NY) with 20% fetal bovine serum (Thermo Fisher Scientific, Waltham, MA) in 5-ml flow tube and chilled at 4°C via water recirculator.

Histologic assessment and immunohistochemistry

Hematoxylin and eosin-stained tissue biopsy slides and corresponding immunohistochemistry were reviewed. Immunohistochemistry for T-cell markers were performed on tissue samples of PTCL as follows; CD2 (MRQ11, Cell Marque, Rocklin, CA), CD3 (LN10, Leica Biosystems, Buffalo Grove, IL), CD4 (SP35, Cell Marque), CD5 (SP19, Ventana, Oro Valley, AZ), CD7 (MRQ56, Cell Marque), CD8 (SP57, Ventana), PD-1 (NAT105, Cell Marque), CD10 (SP67,

Ventana), BCL6 (PG-B6p, Agilent, Santa Clara, CA), and CXCR13 (53610, R&D Systems, Minneapolis, MN). For non-AITL PTCL cases, PD-1 immunohistochemistry was performed on selected cases. Immunohistochemistry for CD20 (L26, Agilent) and CD138 (MI15, Agilent) were used to enumerate B-cells and plasma cells, respectively.

Molecular Analysis

Next generation sequencing to detect AITL-related genetic mutation was performed on formalin fixed paraffin embedded (FFPE) tissue in selected AITL cases. DNA isolation, sequencing library preparation, and sequencing on a HiSeq 2500 were performed as described previously.[6] Hybridization capture was performed using the MSK-IMPACT HEME targeted panel including 400 genes (Suppl. Table 4). All samples from study participants had a matching germline sample. For selected cases, droplet digital PCR (ddPCR) was performed on flow sorted PD-1 bright T-cell population in peripheral blood and bone marrow samples to evaluate the clonal relationship with the tissue sample.

Statistical Analysis

Statistical analysis was performed using the GraphPad Prism 7 (La Jolla, CA). The Student t-test was used for the analysis between 2 groups, and receiver operating characteristic (ROC) curve was created to evaluate the sensitivity and specificity of the assay. A P value of <0.05 was considered statistically significant.

References:

1. Beck RC, et al: Detection of mature T-cell leukemias by flow cytometry using anti-T-cell receptor V beta antibodies. Am J Clin Pathol 120:785-94, 2003

2. Langerak AW, et al: Molecular and flow cytometric analysis of the Vbeta repertoire for clonality assessment in mature TCRalphabeta T-cell proliferations. Blood 98:165-73, 2001

3. Morice WG, et al: Flow cytometric assessment of TCR-Vbeta expression in the evaluation of peripheral blood involvement by T-cell lymphoproliferative disorders: a comparison with conventional T-cell immunophenotyping and molecular genetic techniques. Am J Clin Pathol 121:373-83, 2004

4. Tembhare P, et al: Flow cytometric immunophenotypic assessment of T-cell clonality by Vbeta repertoire analysis: detection of T-cell clonality at diagnosis and monitoring of minimal residual disease following therapy. Am J Clin Pathol 135:890-900, 2011

5. Wu D, Wood BL, Fromm JR: Flow Cytometry for Non-Hodgkin and Classical Hodgkin Lymphoma, in R. K (ed): Lymphoma. Totowa, NJ, Humana Press, 2013, pp 27-41

6. Cheng DT, et al: Memorial Sloan Kettering-Integrated Mutation Profiling of Actionable Cancer Targets (MSK-IMPACT): A Hybridization Capture-Based Next-Generation Sequencing Clinical Assay for Solid Tumor Molecular Oncology. J Mol Diagn 17:251-64, 2015

Supplemental Table 1. Cases tested for PD-1 expression by flow cytometry

	AITL	ALCL ALK-negative	ALCL ALK-positive	ATLL	PTCL - NOS	MF with nodal involvement	T-LGL	T-PLL	NLPHL	THRLBCL	DLBCL, GCB	FL	CHL	Reactive follicular hyperplasia
Total # of patients	36	6	2	9	11	6	5	6	4	1	5	6	12	12
Tissue	28	5	2	3	10	6	0	0	4	1	5	6	12	12
Lymph node	26	3	1	1	6	6	0	0	4	0	0	6	12	9
Spleen	1	0	0	0	1	0	0	0	0	0	0	0	0	0
Bone	0	1	1	0	1	0	0	0	0	0	0	0	0	0
Others	1	1	0	2	2	0	0	0	0	1	0	0	0	3
Peripheral blood	22	2	0	8	5	6	5	6	1	1	0	1	1	1
Bone marrow aspirate	12	0	0	0	0	0	0	0	0	0	3	3	3	0

AITL: Angioimmunoblastic T-cell lymphoma, ALCL: Anaplastic large-cell lymphoma, ATLL: Adult T-cell leukemia/ lymphoma, MF: Mycosis fungoides,PTCL-NOS: Peripheral T-cell lymphoma not otherwise specified

T-LGL: T-cell large granular lymphocytic leukemia, T-PLL: T-cell prolymphocytic leukemia, NLPHL: Nodular lymphocyte-predominant Hodgkin lymphoma, THRLBCL: T-cell/histiocyte-rich large B-cell lymphoma

DLBCL,GCB: Diffuse large B-cell lymphoma, germinal center B-cell type, FL: Follicular lymphoma, CHL: Classical Hodgkin lymphoma

Supplemental Table 2. Immunohistochemistry and flow cytometry findings of tissue biopsy from 28 patients with angioimmunoblastic T-cell lymphoma

Case #	Specimen	Diagnosis	Disease status	History of other LPD	Immunohistochemistry					Abnormal T-cell population	Immunophenotype by flow cytometry						
					CD3	PD-1	CD10	BCL6	CXCL13	by flow cytometry (%)	CD2	sCD3	CD4	CD5	CD7	CD8	CD279 (PD-1)
1	LN, inguinal	AITL	New diagnosis	No	pos	pos	neg	subset pos	ND	8.80	pos	pos	pos	dim	subset neg	neg	pos
2	LN, inguinal	AITL	New diagnosis	No	pos	pos	neg	subset pos	subset pos	21.70	pos	neg	pos	pos	neg	neg	pos
3	LN, axilla	AITL	New diagnosis	No	pos	pos	neg	neg	ND	0.63	pos	neg	pos	pos	pos	neg	pos
4	LN, retroperitoneum	AITL	New diagnosis	No	pos	pos	pos	pos	subset pos	1.10	pos	neg	pos	pos	pos	neg	pos
5	LN, cervical	AITL	New diagnosis	No	pos	pos	pos	ND	ND	14.00	pos	dim	pos	pos	neg	neg	pos
6	LN, inguinal	AITL	New diagnosis	No	pos	pos	subset pos	ND	ND	9.60	pos	neg	pos	pos	neg	neg	pos
7	LN, cervical	AITL	New diagnosis	No	pos	pos	neg	ND	ND	5.20	pos	neg	pos	pos	pos	dim	pos
8	LN, cervical	AITL	New diagnosis	DLBCL	pos	pos	neg	neg	ND	6.70	pos	dim	pos	pos	pos	subset pos	pos
9	LN, inguinal	AITL	New diagnosis	No	pos	pos	neg	subset pos	neg	1.20	pos	pos	pos	pos	neg	neg	pos
10	LN, axilla	AITL	New diagnosis	DLBCL	pos	pos	subset pos	ND	ND	35.30	dim	subset pos, subset neg*	pos	pos	subset neg	neg	pos
11	LN, axilla	AITL	New diagnosis	B-LPD	pos	pos	pos	ND	ND	4.80	pos	neg	pos	pos	pos	neg	pos
12	Soft tissue, base of tongue	AITL	New diagnosis	No	pos	pos	neg	neg	ND	28.60	pos	subset pos, subset neg*	pos	pos	subset neg	neg	pos
13	LN, cervical	AITL	New diagnosis	CHL	pos	pos	neg	subset pos	neg	10.70	pos	neg	pos	pos	pos	neg	pos
14	LN, cervical	AITL	New diagnosis	No	pos	pos	pos	ND	ND	22.20	pos	neg	pos	pos	pos	neg	pos
15	LN, cervical	AITL	Relapse/ persistent	No	pos	pos	neg	ND	ND	7.80	pos	dim	pos	pos	neg	neg	pos
16	LN, axilla	AITL	Relapse/ persistent	DLBCL	pos	pos	subset pos	ND	ND	0.75	pos	neg	pos	pos	subset neg	neg	pos
17	LN, axilla	AITL	Relapse/ persistent	No	pos	pos	pos	subset pos	ND	19.10	pos	subset pos, subset neg*	pos	pos	subset neg	neg	pos
18	Spleen	AITL	Relapse/ persistent	No	pos	pos	subset pos	ND	subset pos	14.60	pos	neg	pos	pos	pos	neg	pos
19	LN, axilla	AITL	Relapse/ persistent	No	pos	pos	neg	ND	ND	27.80	pos	pos	dim	dim	pos	neg	pos
20	LN, neck	AITL	Relapse/ persistent	No	pos	pos	pos	ND	ND	2.90	pos	neg	pos	pos	subset neg	neg	pos
21	LN, cervical	AITL and DLBCL, GCB type	Relapse/ persistent	DLBCL, GCB type	pos	pos	NC	NC	ND	0.12	pos	neg	pos	pos	pos	neg	pos
22	LN, axilla	AITL and CD8-positive T-LPD	Relapse/ persistent	T-LPD	pos	pos	subset pos	ND	ND	0.19	pos	pos	pos	pos	neg	neg	pos
23	LN, axilla	AITL	Relapse/ persistent	No	pos	pos	neg	subset pos	ND	26.90	pos	subset pos, subset neg*	pos	pos	dim	neg	pos
24	LN, inguinal	AITL	Relapse/ persistent	No	pos	pos	ND	neg	subset pos	88.30	pos	dim	dim	pos	neg	neg	pos
25	LN, inguinal	AITL	Relapse/ persistent	No	pos	pos	neg	pos	neg	17.80	pos	neg	subset pos	pos	pos	neg	pos
26	LN, inguinal	PTCL-TFH	Relapse/ persistent	No	pos	pos	neg	subset pos	ND	90.60	pos	pos	dim	neg	neg	neg	pos
27	LN, axilla	AITL an B-LPD	Relapse/ persistent	B-LPD	pos	pos	pos	subset pos	ND	8.50	pos	dim	dim	pos	neg	neg	pos
28	LN, axilla	AITL	Relapse/ persistent	No	pos	pos	neg	neg	neg	22.80	pos	neg	pos	pos	neg	neg	pos

AITL: Angioimmunoblastic T-cell lymphoma, CHL: Classical Hodgkin Lymphoma, DLBCL: Diffuse large B-cell lymphoma,LN: Lymph node, LPD: Lymhoproliferative disorder, NC: Non contributory, ND: Not done

*: sCD3 positive population confirmed as clonal by Vbeta analysis

Supplemental Table 3. Molecular findings of 14 cases of angioimmunoblastic T-cell lymphoma evaluated with formalin-fixed parrafin enbedded tissue

Case #	Molecular findings				
	**RHOA**	**TET2**	**IDH2**	**DNMT3A**	**Other mutated genes**
3	WT	Q909fs, C1378F	WT	WT	WT
4	G17V	C973fs, R1216*	R172S	WT	WT
5	G17V	R1261C, TET2 D1704Mfs*15	WT	WT	WT
6	WT	Q1537*, N1610Ifs*6	WT	I681M	WT
7	WT	C1289Y, L1899Sfs*9,	WT	R771*	SMO L22
10	WT	F381fs, R550*	WT	WT	HIST1H3D, NOTCH1, REL, SOCS1, SPEN
11	WT	N1266fs, I1873T	WT	WT	EGFR
12	G17V	C1273Y, I274fs	WT	WT	WT
14	G17V	R1359S	WT	Q249*	WT
19	WT	P555Hfs*6, P1894L	WT	WT	FAS, PAX5, SOCS1, VAV1
21	G17V	R1167K, V1900del	R172M	exon15 splicing variant	WT
22	WT	Y592fs	WT	exon11 splicing variant	CHEK1, ROS1
24	C16F	WT	WT	WT	TP53, CDKN2Ap16INK4A, CDKN2Ap14ARF, CDKN2B, FAT1, ROBO1
25	WT	Q531*, W954*, F1368V	WT	R882C	WT

WT: Wild type

Supplemental Table 4. MSK-IMPACT HEME 400 gene panel

ABL1	BIRC3	CTNNB1	FLCN	ID3	MCL1	PAK7	REL	SPEN
ACTG1	BLM	CUX1	FLT1	IDH1	MDM2	PALB2	RET	SPOP
AKT1	BRAF	CXCR4	FLT3	IDH2	MDM4	PARP1	RHOA	SRC
AKT2	BRCA1	CYLD	FLT4	IGF1	MED12	PAX5	RICTOR	SRSF2
AKT3	BRCA2	DAXX	FOXL2	IGF1R	MEF2B	PBRM1	RNF43	STAG1
ALK	BRD4	DDR2	FOXO1	IGF2	MEN1	PCBP1	ROBO1	STAG2
ALOX12B	BRIP1	DDX3X	FOXP1	IKBKE	MET	PDCD1	ROS1	STAT3
AMER1	BTG1	DIS3	FURIN	IKZF1	MGA	PDGFRA	RPTOR	STAT5A
APC	BTK	DNMT3A	FYN	IKZF3	MGAM	PDGFRB	RRAGC	STAT5B
AR	CALR	DOT1L	GATA1	IL7R	MITF	PDPK1	RTEL1	STAT6
ARAF	CARD11	DTX1	GATA2	INPP4B	MLH1	PDS5B	RUNX1	STK11
ARHGEF28	CASP8	DUSP22	GATA3	IRF1	MOB3B	PHF6	RUNX1T1	SUFU
ARID1A	CBFB	EED	GNA11	IRF4	MPEG1	PIGA	SAMHD1	SUZ12
ARID1B	CBL	EGFR	GNA12	IRF8	MPL	PIK3C2G	SDHA	SYK
ARID2	CCND1	EGR1	GNA13	IRS2	MRE11A	PIK3C3	SDHB	TBL1XR1
ARID3A	CCND2	EP300	GNAQ	JAK1	MSH2	PIK3CA	SDHC	TBX3
ARID3B	CCND3	EP400	GNAS	JAK2	MSH6	PIK3CG	SDHD	TERT
ARID3C	CCNE1	EPHA3	GNB1	JAK3	MTOR	PIK3R1	SETBP1	TET1
ARID4A	CD274	EPHA5	GRIN2A	JARID2	MUTYH	PIK3R2	SETD1A	TET2
ARID4B	CD28	EPHA7	GSK3B	JUN	MYC	PIM1	SETD1B	TET3
ARID5A	CD58	EPHB1	GTF2I	KDM5A	MYCL1	PLCG1	SETD2	TGFBR2
ARID5B	CD79A	ERBB2	HDAC1	KDM5C	MYCN	PLCG2	SETD3	TNFAIP3
ASXL1	CD79B	ERBB3	HDAC4	KDM6A	MYD88	PMS2	SETD4	TNFRSF14
ASXL2	CDC73	ERBB4	HDAC7	KDR	NBN	PNRC1	SETD5	TOP1
ATM	CDH1	ERG	HGF	KEAP1	NCOR1	POT1	SETD6	TP53
ATP6AP1	CDK12	ESCO2	HIF1A	KIT	NCOR2	PPP2R1A	SETD7	TP63
ATP6V1B2	CDK4	ESR1	HIST1H1B	KMT2A	NCSTN	PRDM1	SETD8	TRAF2
ATR	CDK6	ETNK1	HIST1H1C	KMT2B	NF1	PRKAR1A	SETDB1	TRAF3
ATRX	CDK8	ETV6	HIST1H1D	KMT2C	NF2	PTCH1	SETDB2	TRAF5
ATXN2	CDKN1B	EZH2	HIST1H1E	KMT2D	NFE2	PTEN	SF3B1	TSC1
AURKA	CDKN2A	FAM46C	HIST1H2AC	KRAS	NFE2L2	PTPN1	SGK1	TSC2
AURKB	CDKN2B	FANCA	HIST1H2AG	KSR2	NKX2-1	PTPN11	SH2B3	TSHR
AXIN1	CDKN2C	FANCC	HIST1H2AL	LCK	NOTCH1	PTPN2	SMAD2	TYK2
AXL	CEBPA	FANCD2	HIST1H2AM	LMO1	NOTCH2	RAD21	SMAD4	U2AF1
B2M	CHEK1	FAS	HIST1H2BC	LTB	NOTCH3	RAD50	SMARCA4	U2AF2
BACH2	CHEK2	FAT1	HIST1H2BD	MALT1	NOTCH4	RAD51	SMARCB1	UBR5
BAP1	CIC	FBXO11	HIST1H2BG	MAP2K1	NPM1	RAD51B	SMARCD1	VAV1
BARD1	CIITA	FBXW7	HIST1H2BJ	MAP2K2	NRAS	RAD51C	SMC1A	VAV2
BCL10	CRBN	FGF19	HIST1H2BK	MAP2K4	NSD1	RAD51D	SMC3	VHL
BCL11B	CREBBP	FGF3	HIST1H2BO	MAP3K1	NT5C2	RAD52	SMG1	WHSC1
BCL2	CRKL	FGF4	HIST1H3B	MAP3K13	NTRK1	RAD54L	SMO	WT1
BCL6	CRLF2	FGFR1	HIST1H3G	MAP3K14	NTRK2	RAF1	SOCS1	XBP1
BCOR	CSF1R	FGFR2	HLA-A	MAPK1	NTRK3	RARA	SOX2	XPO1
BCORL1	CSF3R	FGFR3	HNF1A	MAPK3	P2RY8	RB1	SP140	ZRSR2
BCR	CTCF	FGFR4	HRAS					

Supplemental Figure 1

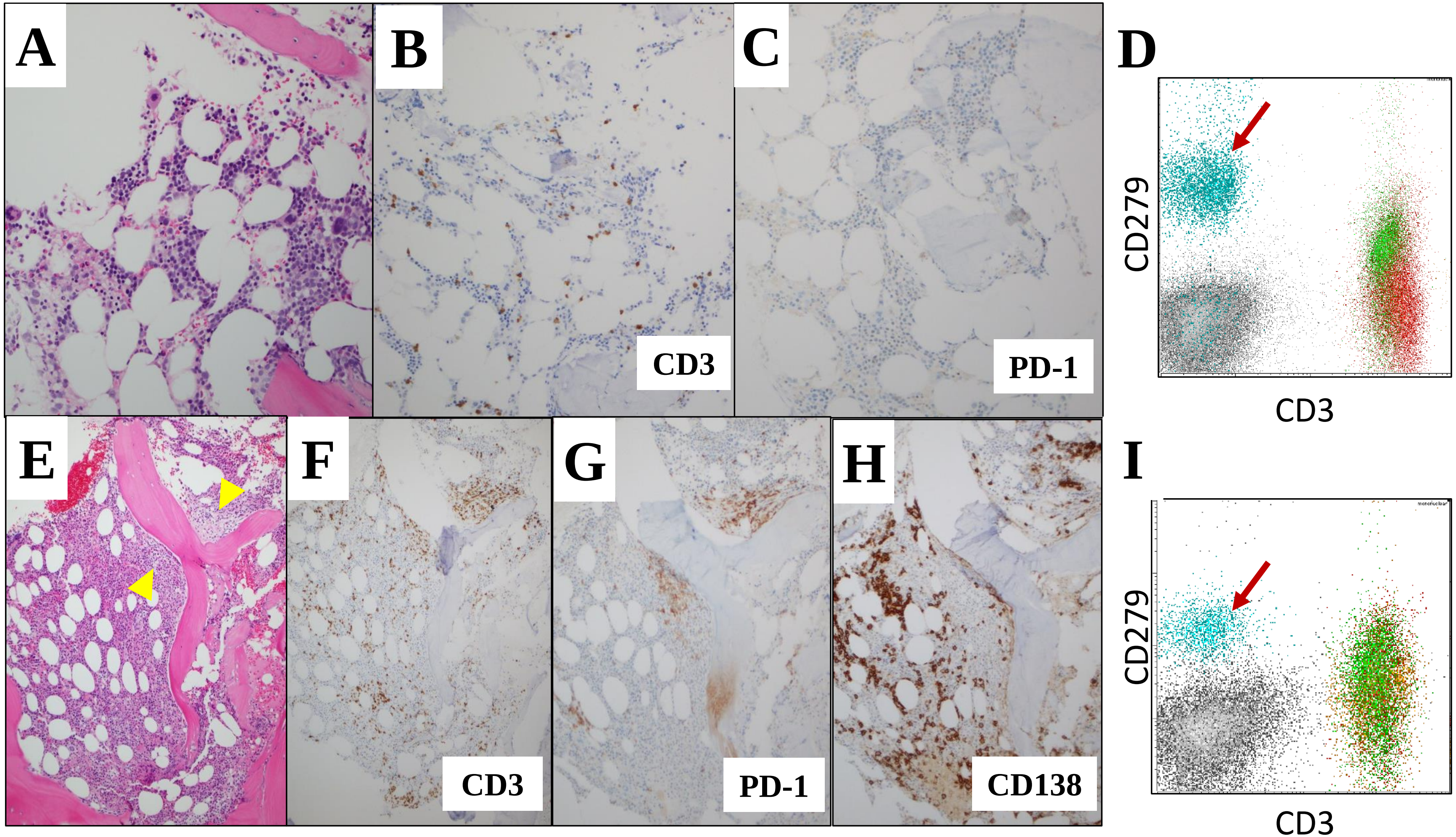

Supplemental Figure 1. Bone marrow biopsy of the patients with AITL. Bone marrow shows normocelluar marrow with trilineage hematopoiesis (A). CD3 immunohistochemistry highlights scattered T-cells (B). PD-1 immunohistochemistry highlights scattered dimly positive cells; however, interpretation is difficult (C). Flow cytometry detects abnormal T-cell population with CD3-/CD4+/PD-1 bright immunophenotype (arrow), consistent with involvement by AITL, 1% of total white blood cells by flow cytometry (D). Atypical paratrabecular lymphoid aggregates (arrow head) are present (E). These atypical lymphocytes express CD3 (F) and PD-1 (G). This bone marrow also shows concomitant plasma cell neoplasm (H). Flow cytometry detects abnormal T-cell population with CD3-/CD4+/PD-1 bright immunophenotype (arrow), consistent with involvement by AITL, 0.51% of total white cells by flow cytometry (I).

A: hematoxylin and eosin, x200; B: CD3 immunohistochemistry, x200, C: PD-1 immunohistochemistry, x200; E: hematoxylin and eosin, x100; F: CD3 immunohistochemistry, x100, G: PD-1 immunohistochemistry, x100; H: CD138 immunohistochemistry, x100

Supplemental Figure 2

Abnormal T-cell population with sCD3+/ CD4+/ CD279 bright immunophenotype, showing Vbeta restriction (25.4% of total WBC)

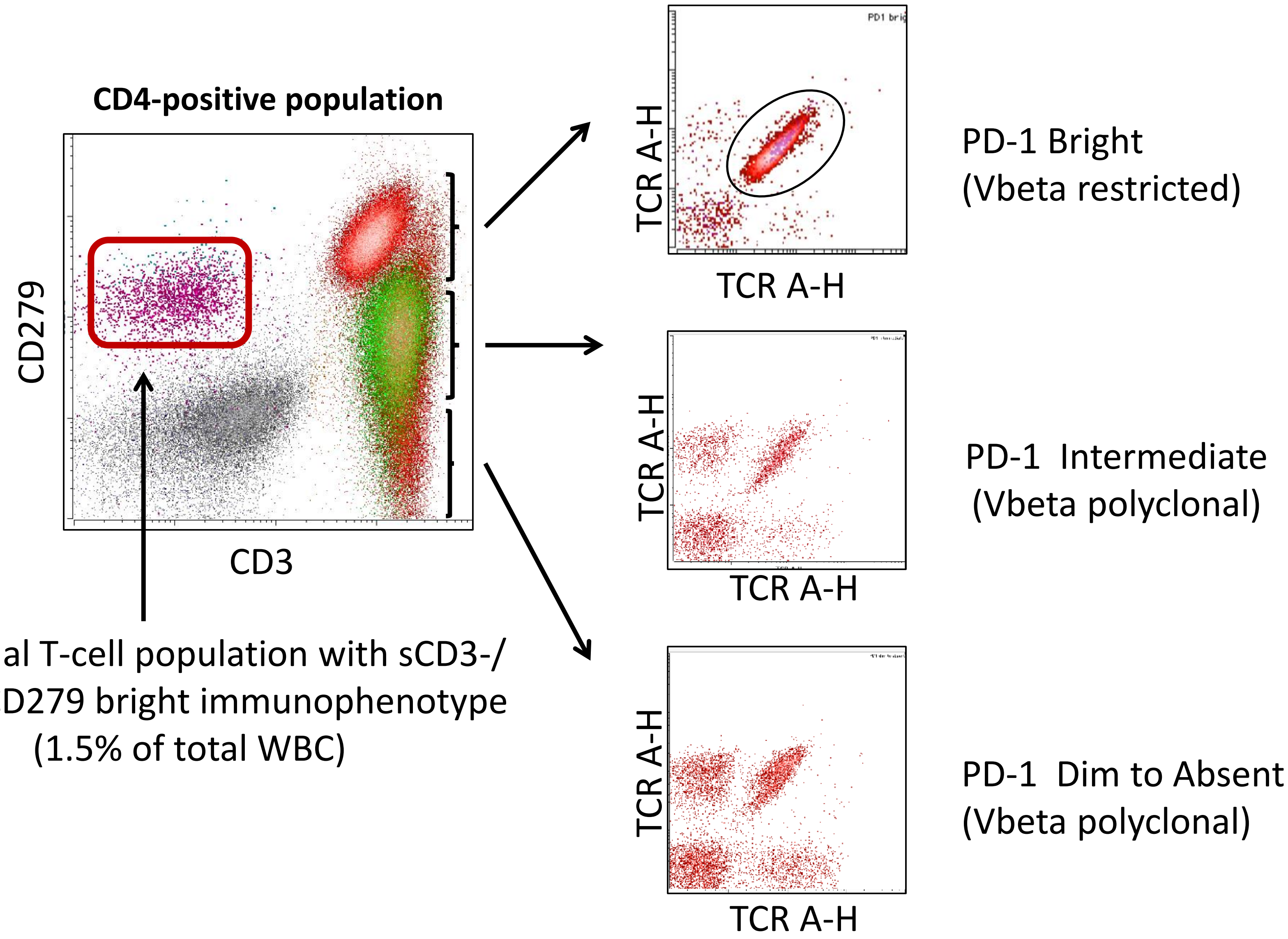

Abnormal T-cell population with sCD3-/ CD4+/ CD279 bright immunophenotype (1.5% of total WBC)

Supplemental Figure 2. Flow cytometry showing both sCD3+ and sCD3- AITL populations (Case 23). In addition to PD-1 bright population with aberrant loss of sCD3 (sCD3-/ CD4+/ CD279 bright; 1.5% of total WBC), PD-1 bright population expressing sCD3 was also demonstrated as clonal by Vbeta fragment analysis after flow sorting (25.4% of total WBC).
